# Capsaicin orchestrates metastasis in gastric cancer via modulating expression of TRPV1 channels and driving gut microbiota disorder

**DOI:** 10.1186/s12964-023-01265-3

**Published:** 2023-12-21

**Authors:** Rui Deng, Suyun Yu, Xingqiu Ruan, Huan Liu, Gangfan Zong, Peng Cheng, Ruizhi Tao, Wenxing Chen, Aiyun Wang, Yang Zhao, Zhonghong Wei, Yin Lu

**Affiliations:** 1 Jiangsu Key Laboratory for Pharmacology and Safety Evaluation of Chinese Materia Medica, School of Pharmacy, Nanjing University of Chinese Medicine, Nanjing, 210023 China; 2 State Key Laboratory Cultivation Base for Traditional Chinese Medicine (TCM) Quality and Efficacy, Nanjing University of Chinese Medicine, Nanjing, 210023 China; 3 Red Cross Hospital of Yulin City, Yulin, 537000 China; 4 School of Medicine & Holistic Integrative Medicine, Nanjing University of Chinese Medicine, Nanjing, 210023 China; 5 Jiangsu Collaborative Innovation Center of Traditional Chinese Medicine (TCM) Prevention and Treatment of Tumor, Nanjing University of Chinese Medicine, Nanjing, 210023 China

**Keywords:** High capsaicin diet, Gastric cancer, Gut microbiota, TRPV1 channel, 5-HT

## Abstract

The association between capsaicin, the major natural pungent compound of chili peppers, and gastric cancer progression has engendered conflicting findings. In this work, we sought to explore the character of a high capsaicin diet in gastric cancer metastasis and its possible mechanism. The impact of high capsaicin consumption on gastric cancer metastasis was investigated in vivo (xenograft mouse and zebrafish models) and in vitro (biochemical and molecular assays). It was demonstrated that high diet of capsaicin gave rise to accelerate tumor metastasis, which was partially mediated by elevating the expression of transient receptor potential vanilloid 1 (TRPV1) in gastric cancer cells. Importantly, we found that genetic depletion of TRPV1 could reduce gastric cancer metastasis by diminishing the motility of tumor cells in vitro, but acted poorly in xenograft mouse model. Considering the distribution of capsaicin in vivo, 16*S* rRNA sequencing and fecal microbiota transplantation (FMT) were used to appraise whether the gut microbiota involved in the high capsaicin diet induced metastasis. It was demonstrated that the level of *Firmicutes* and *Clostridiales* was expressively boosted following the high consumption of capsaicin. This microbial shift contributed to the increased peripheral 5-hydroxytryptamine (5-HT) levels, yielding the aggravated metastatic burden. Collectively, our findings highlighted the potential risk of high capsaicin diet in promoting gastric cancer metastasis by virtue of modulating TRPV1 expression and gut microbiota composition, indicating the importance of controlled consumption of chili peppers for patients with gastric cancer.

Video Abstract

**Supplementary Information:**

The online version contains supplementary material available at 10.1186/s12964-023-01265-3.

## Introduction

Gastric cancer (GC) is globally acknowledged as one of the primary causes of cancer-related mortality [1]. A large population of GC patients is diagnosed at a locally advanced or metastatic stage, frequently presenting with the development of lymphatic and peritoneal metastases [2]. Currently, surgical resection remains the sole potential curative treatment offered in clinical practice. The widespread occurrence of metastatic GC has imposed a substantial healthcare burden worldwide, particularly in East Asian countries [3]. The prognosis of patients with advanced metastatic GC is extremely poor, but early diagnosis and treatment can significantly improve survival rates [3]. Notably, it has been widely held that inappropriate diet habit is a risk factor contributing to the progression of GC [4]. Consequently, the effective treatment of GC remains challenging, partly due to the limited understanding of the role of diet in influencing GC metastasis.

Chili peppers have been widely employed in culinary practices and account for a substantial proportion of vegetables consumed every day around the world. The pungency, intensity, and hotness of chili peppers are primarily attributed to capsaicin [5]. According to the data of 2002 from European Commission Health & Consumer Protection Directorate-General, the average daily consumption of capsaicin was estimated to range from 25 to 200 mg in Thailand and Mexico [6]. Remarkably, statistical analysis reveals that Mexican adults who consumed 30–250 mg or 90–250 mg of capsaicin per day, as well as Korean adults whose daily intake amounted to 8.10–19.44 mg, can be classified as members of high capsaicin dietary groups [7–10]. A growing body of epidemiological studies carried out in different countries (including, China, India, Mexico and Tanzania) have established a positive correlation between the high consumption of chili peppers or capsaicin and an increased risk of tumor progression [7, 11–15]. Recent researches have also demonstrated a noteworthy role of capsaicin in promoting tumor metastasis, particularly in lung cancer and GC [16, 17]. Despite these findings, the precise mechanisms underlying the influence of a high capsaicin diet on GC metastasis remain elusive.

It has been reported that the gastrointestinal tract can absorb approximately 85% of the orally administrated capsaicin, which is subsequnetly transported to the portal vein within a three-hour timeframe [18]. Importantly, high-dose capsaicin (80 mg/kg) tends to disrupt the intestinal barrier, alter the gut microbiota composition, and induce gastric inflammation [19]. Interestingly, recent findings have shown that gut microbiota is involved in regulating the biosynthesis of 5-HT (5-hydroxytryptamine, serotonin), which is predominantly derived from colonic enterochromaffin cells [20]. Additionally, it has also been uncovered that autocrine 5-HT from triple-negative breast cancer (TNBC) cells is essential for accelerating the progression of TNBC through interacting with the 5-HT7 receptor [21]. Furthermore, heightened expression of 5-HT1D has been demonstrated in the tissues of hepatocellular carcinoma (HCC) compared to normal tissue, and this upregulation is positively correlated with poor prognosis and shortened overall survival (OS) of HCC patients [22].

TRPV1 is a non-selective cation ligand-gated channel with high permeability for calcium ion (Ca^2+^). It was the first identified and the most extensively studied member of the TRPV subfamily. As the only member of the TRPV subfamily which can be activated through capsaicin, TRPV1 has been defined as a receptor of capsaicin [23]. Upon activation, TRPV1 facilitates the influx of extracellular Ca^2+^ into cells, thereby triggering the activation of PI3K/Akt/mTOR signaling pathway [24]. Beyond its physiological role in normal cells, the contribution of TRPV1 to tumor progression has also been widely investigated [25]. For instance, TRPV1 is highly expressed in human colorectal cancer and related with tumor progression and inferior survival [26]. The protein expression of TRPV1 is upregulated in the esophageal squamous cell carcinoma (ESCC) cells, and the recurrent activations of TRPV1 promotes the migration of ECA109 cells [27]. However, the role of TRPV1 in GC progression remains elusive.

In the present study, we demonstrated that a high capsaicin diet exacerbated metastasis in mice with GC. Mechanistic studies unravelled that the accelerated metastatic process induced by capsaicin was partially mediated by the upregulation of TRPV1 in the gastric cancer cells. Moreover, high-level consumption of capsaicin resulted in gut microbiota disorder, particularly manifested as alterations in the abundance of *Firmicutes* and *Clostridiales*, which further gave rise to the increased levels of peripheral 5-HT. The interactions between 5-HT and its receptor HTR3A exerted a striking effect on propelling metastasis in GC. Taken together, our findings highlighted the role of a high capsaicin diet in influencing GC metastasis, which might also provide a new perspective to offer a potential and promising therapeutic strategy for gastric cancer.

## Methods

### Reagents and cells

Lipofectamine 2000 reagents (11,668–019) were purchased from Invitrogen (Massachusetts, USA). Human GC cell lines (AGS, BGC-823, MKN-45), and human gastric mucosa cell line (GES-1) were as gifts generously given by A/Prof. Yunlong Shan (Key Laboratory of Drug Metabolism and Pharmacokinetics, State Key Laboratory of Natural Medicines, China Pharmaceutical University). AGS, BGC-823, and MKN-45 were cultured in RPMI 1640 (Thermo Fisher, USA) with 10% fetal bovine serum (TIANHANG, Zhejiang, China), 100 U/mL penicillin (CAS NO.113–98-4, Merck, Germany), and 100 μg/mL streptomycin (CAS NO.3810–74-0, Merck, Germany). Cells were sustained in a humidified incubator (37℃, 5% CO_2_). MKN-45-luc cells and BGC-823-luc cells were generated via stable transduction with lentivirus (GENECHEM, Shanghai, China). Firstly, 2 × 10^5^ MKN-45 and BGC-823 cells were seeded into the 6-well plates, 40 μL HiTransG P (GENECHEM, Shanghai, China) and 40 μL lentivirus (1 × 10^8^ TU/mL) were added to co-culture with the cells. After 12 h incubation, the medium containing lentivirus of luciferase expressing MKN-45 and BGC-823 cells were selected with 2 μg/mL of puromycin (Invitrogen, San Diego, USA) for 1 week. Finally, the stable MKN-45-luc and BGC-823-luc cells were cultured in the RPMI-1640 (Gibco, California, USA) with 10% fetal bovine serum (FBS) (Gibco, California, USA), and 0.5 μg/mL of puromycin and incubated at 37 °C, 5% CO_2_. To construct the TRPV1-knockout MKN-45 cell line (TRPV1-KO-MKN-45), we used the pCas-Puro-U6-TRPV1-KO plasmid (Corues Biotechnology, Nanjing, China) and Lipofectamine 2000 reagents (Invitrogen, 11,668–019, Massachusetts, USA), along with Opti-MEM Reduced Serum Medium (Gibco, 21,516,800). Firstly, 5 × 10^4^ MKN-45 cells were seeded in the 24-well plate and maintained in the RPMI-1640 with 10% FBS, without streptomycin or penicillin. 4 μg plasmid and 10 μL Lipofectamine 2000 (Invitrogen, San Diego, USA) were mixed in 500 μL Opti-MEM. 50 μL mixture was added to co-culture with the cells. The medium was removed after 12 h incubation, and the new growth medium supplemented with puromycin (2 μg/mL) was added to select the retrovirus-infected cells. After 72 h, single cell clones of TRPV1 depletion were grown and screened via immunoblotting. From dozens of surviving cells after several days of puromycin treatment, an array of clones was selected and expanded. Finally, the stable TRPV1-MKN-45 cells were cultured in the RPMI-1640 with 10% FBS, and 0.5 μg/mL of puromycin and incubated at 37 °C, 5% CO_2_. The sequence of the TRPV1 plasmid is shown in Table S1.

### Capsaicin diet preparation

Based on previous reports, a high capsaicin diet has been defined as an adult intake of 90–250 mg of capsaicin per day [7–10]. Considering the bias of different calculation methods, we chose 90 or 200 mg/day as the minimum or maximum dose for high capsaicin diet, which is equivalent to 1.29/2.86 mg/kg body weight (BW) (an adult at 70 kg), 11.7/26 mg/kg BW (a mouse at 20 g). According to the observations of the previous experiments, each mouse ingested about 5 g of feeds per day and the amount of capsaicin added to the feed was further estimated to be 46.8/104 mg/kg. Therefore, two doses of capsaicin diet (50 mg/kg and 100 mg/kg) were applied in this experiment. Briefly, capsaicin (CAS NO.404–86-4, HPLC ≥ 98%) obtained from Yuanye (Shanghai, China) was added into the basal diet at 0, 0.005, and 0.01% supplementation by Jiangsu Xietong Pharmaceutical Bio-engineering Co., LTD (Nanjing, China).

### Xenograft mouse tumor models

The BALB/c nude mice (6-weekold, male) were purchased from Shanghai SLAC Laboratory Animal Co., LTD (Shanghai, China). The mice were kept under controlled laboratory conditions with standard 12 h/12 h light/dark cycle (*n* = 3–5 per cage). Experiments on mice were conducted according to the Guidelines for the Care and Use of Laboratory Animals and approved by the Animal Committee of Nanjing University of Chinese Medicine (permission no. 202005A019).

For the establishment of cell line-derived xenograft (CDX) model, BALB/c nude mice were anesthetized by exposure to isoflurane and then a 1 cm incision was carefully made in the abdomen using sterile scissors. Suspensions of MKN-45 cells, BGC-823 cells, and TRPV1-KO-MKN-45 cells at a concentration of 1 × 10^7^/mL in sterilized PBS were injected into the epidermis of the greater curvature of the stomach, with 100 µL cell suspension per mouse. The injection site was pressed with a cotton swab for 1 min to prevent the cell suspension leakage. Subsequently, the wound was sterilized with iodine, and the implant area was closed by subcuticular sutures with surgical needles [28]. For the metastasis model, 100 µL MKN-45 cells at the concentration of 5 × 10^6^/mL in sterilized PBS were intravenously injected into the mice. The next day, the mice were randomly divided into three groups including the model group, 50 mg/kg capsaicin diet group, and 100 mg/kg capsaicin diet group. The mice were maintained with either regular diet or diets containing 50 mg/kg or 100 mg/kg capsaicin.

### In vivo bioluminescence imaging

According to the established protocol, in vivo bioluminescence imaging was processed with minor modifications [29]. For bioluminescence imaging of MKN-45-luc and BGC-823-luc tumor-bearing mice, D-luciferin (15 mg/mL stock solution, 200 μL/mice, Gold Biotechnology) was inoculated into mice by the retro-orbital venous plexus using 30G needles. Bioluminescence images were collected by IVIS Spectrum Imaging System (PerkinElmer, Waltham, USA) at 5 min post injections.

### Zebrafish xenograft model

The Tg (fli1:EGFP) transgenic zebrafish line, which expresses enhanced green fluorescent protein (EGFP) in the vascular endothelium, was obtained from Nanjing YSY Biotech. Co., LTD (Nanjing, China). Zebrafish were maintained and raised in a professional zebrafish facility at the laboratory. Experiments on zebrafish embryos were reviewed and approved by the Animal Committee of Nanjing University of Chinese Medicine (permission no. 20210116). Zebrafish embryos were gathered 48 h postfertilization (48 hpf) and dechorionated using 1 mg/ml pronase E. After dechorionation, embryos were anesthetized with 0.6 mM tricaine and placed on a transparent dish coated with 1.5% agarose for the subsequent injection. Dil-labelled MKN-45 cells were then microinjected into the yolk sac of embryos (250 cells per embryo) using a microinjector (WPI, Stevenage, UK). The next day, the live embryos were exposed to 16 μM capsaicin and then incubated at 28 ± 1 ℃ for 24 h. The number of tumor cells was quantified based on fluorescent intensity using a Leica Thunder Imaging System.

### Cell proliferation assays

DMSO was used as a solvent to prepare capsaicin stock solution at 256 mM. Then we used DMSO to gradient dilute the 256 mM capsaicin stock solution to get 128 mM, 64 mM, 32 mM, 16 mM, 8 mM, 4 mM, and 2 mM capsaicin solutions. In the experiments, the final concentration of capsaicin we used was 256 μM, 128 μM, 64 μM, 32 μM, 16 μM, 8 μM, 4 μM, and 2 μM. In the control group, we added 0.1% DMSO to PBS to eliminate the effect of DMSO on cell viability. The proliferation abilities of cells were assessed by Cell Counting Kit-8 (CCK-8) assay and 5-Ethynyl-2-deoxyuridine (EdU) assay. For the CCK-8 assay, cells were plated into 96-well plates. After the 24 h treatment of capsaicin, CCK-8 (CT01C, Cellcock, Guangzhou, China) was added to the 96-well plates and incubated at 37℃ for 2 h. Subsequently, optical density values at 450 nm were measured using a microplate reader (Synergy2, BioTek, Burlington, USA). In terms of EdU assay, the proliferation of cells was performed using an EdU kit (C0075S, Beyotime, Shanghai, China) according to the manufacturer’s instructions. The EdU-positive cells were analyzed using a fluorescence microscope (Axio vert A1, ZEISS, Oberkochen, Germany) and the percentage of EdU-positive cells was calculated based on three random fields.

### Transwell migration assay

MKN-45, TRPV1-KO-MKN-45 and BGC-823 cells treated with or without capsazepine (CAS NO.138977–28-3, MedChemExpress, New Jersey, USA) for 30 min were seeded onto the transwell inserts (3422, Corning, New York, USA). The filter insert was then transferred onto a 24-well plate, in which culture medium contained 16 μM capsaicin. After incubation at 37℃ for 24 h, the migrated cells on the lower face of the polycarbonate membrane were fixed with 4% paraformaldehyde (PFA) and stained with 1% crystal violet (C0121, Beyotime, Shanghai, China). The migrated cells were counted under a bright-field microscope (Axio vert A1, ZEISS, Oberkochen, Germany) and three random fields of each chamber were selected for further quantification.

### Microfluidic migration assay

Microfluidic devices containing arrays of parallel microchannels with a width of 3 μm dimensions were used for cell migration assay. The migration of cells was induced by the gradient of serum. The live cell imaging analysis system was set to collect an image every 15 min for 4 h. The distance of cell migration was recorded using a fluorescence microscope (Axio vert A1, ZEISS, Oberkochen, Germany).

### 3D cell invasion assay

The invasion of cells was measured using a 3D culture system. In brief, MKN-45, TRPV1-KO-MKN-45 and BGC-823 cells were suspended with RPMI 1640 medium and matrigel (354,234, Corning, New York, USA) mixture (2:1). 1 μL cell suspension was carefully plated into a 15 mm glass-bottom confocal dish (801,002, NEST, Wuxi, China). Upon solidification of cell suspension, 250 μL RPMI 1640 medium and matrigel mixture (2:1) was affiliated to seal the matrix. Then RPMI 1640 medium containing different concentrations of capsaicin were used to overlay the setup after polymerization. The morphology of the 3D culture system was captured by a bright-field microscope as the original image. After cells invaded the outer matrix for 24 h, a second shot of the system was taken. Images at 0 h and 24 h were overlaid by Adobe Illustrator CS6 to analyze the invaded cells.

### Western blot analysis

Cell protein was extracted using a lysis buffer (P0013B, Beyotime, Nanjing, China), which was supplemented with protease and phosphatase inhibitor cocktail (ST506, Beyotime, Nanjing, China; GB-0032, KeyGEN, Nanjing, China). After incubation on ice for 30 min, the lysates were centrifuged at 12,000 rpm for 10 min at 4 ℃ and the supernatants were collected. The total proteins were separated by 6%-10% SDS-PAGE gels, then immediately transferred onto PVDF films (Millipore, Darmstadt, Germany). The films were furtherly probed by primary antibodies overnight (4℃), and then incubated with corresponding secondary antibodies. A BIORAD imaging system (chemiDOCTMXRS, USA) was applied to visualized the protein bands. The primary antibodies used in this study were listed as follows: Anti-TRPV1 (NB100-1617, Novus Biologicals, Colorado, USA), Anti-SR-3A (sc-390168, Santa Cruz, USA), Anti-5HT-H209 (NB120-16007, Novus Biologicals, Colorado, USA), anti-mTOR (2983, CST, Boston, USA), anti-p-mTOR (5536, CST), anti-PI3K (A0265, ABclonal, Wuhan, China) and anti-GAPDH (A19056, ABclonal, Wuhan, China).

### Real-time PCR

In brief, TRIzol reagent (Thermo Fisher Scientific, Waltham, USA) were used to lyse the cell samples and the total RNA was isolated by chloroform and isopropanol. 500 ng cDNA were used to synthesize cDNA using Hiscript®II QRT SuperMix (Vazyme, Nanjing, China). Real-time PCR was implemented (ChamQ SYBR qPCR Master Mix, Vazyme, Nanjing, China) and tested by ABI 7500 system (Applied Biosystems, California, USA). The sequences of primers used in this study are listed in Table S2.

### Detection of capsaicin by ultra-performance liquid chromatography coupled with triple-quadrupole tandem mass spectrometry (UPLC-TQ-MS/MS)

UFLC SIL-20AXR LC in-line system (Shimadzu Corporation, Kyoto, Japan) combined with a QTRAP 5500 system (AB SCIEX) was used for chromatographic analysis and mass identification. Multiple reaction monitoring (MRM) technology was applied for quantitative analysis and a Waters XSelect® CSHTM C18 column XP (100 mm × 3.0 mm, 2.5 μm) was used for chromatographic separation. 4 μL of each sample was injected into the system, and then eluted by mixed mobile phase (0.4 mL/min) comprised with 0.1% formic acid solution (A) and acetonitrile. The following gradient elution program was set: 2.5 min (85% A), 7 min (20% A), 9 min (20% A), 11 min (15%A). The content of capsaicin was quantified by MRM mode (detailed parameters see Table S3). A series of standard solutions (2.6–42.2 ng/mL) were prepared to construct standard calibration curves. MRM parameters of capsaicin by UPLC-TQ-MS and linear regression data are shown in Tables S3 and 4.

### 5-HT measurement by high-performance liquid chromatography with fluorescence detection (HPLC-FLR)

Serum samples (100 μL each) were acidified with 0.2 M perchloric acid (100 μL each). After vortexing (13,000 rpm) for 30 s, the mixture was centrifuged at 4 ℃. 40 μL supernatant was furtherly mixed with 160 μL mobile phase and centrifuged for another 15 min. The final supernatants were injected into a Waters HPLC system equipped with a 2475 FLR detector (Waters, Milford, USA). A Syncronic C18 column and an Agilent Zorbax Extend C18 column were used for chromatographic separation. It was eluted by 0.1 M potassium dihydrogen phosphate and acetonitrile (9:1, v/v) at 1.0 mL/min. Fluorescence detection was performed at λex/λem 280/330 nm.

### Immunofluorescence

The paraffin-embedded sections were deparaffinized using xylene, and then rehydrated by a series concentration of ethanol. Antigen retrieval solution (citrate buffer) was subsequently used. 5% BSA was applied to block the glass slides for 30 min. Two primary antibodies including anti-MAOA (A4105, ABclonal, Wuhan, China) and anti-TPH1 (bs-1215R, Bioss, Massachusetts, USA) were applied to incubate with sections overnight. The next day, suitable secondary antibodies with fluorescent labels were used to incubate for another 1 h at room temperature. Finally, DAPI (C1006, Beyotime, Shanghai, China) was used to counterstained the cell nucleus. A fluorescence inversion microscope (stemi 2000C, Olympus, Miyazaki, Japan) was applied to image the sections. For Ca^2+^ imaging, Fluo-4 AM (S1060, Beyotime, Shanghai, China) was used to trace intracellular calcium. A total of 5 × 10^4^ cells were seeded on coverslips and incubated with Fluo-4 AM probe at 37 °C for 30 min in dark. The fluorescence of Fluo-4 AM probe was measured at 488 nm by a Zeiss fluorescence imaging system. For F-actin staining, Phalloidin-iFluor 488 (ab176753, Abcam, Cambridge, UK) was used to stain F-actin, and DAPI was used to counterstain the cell nucleus.

### ELISA assay

The 5-HT level in the supernatants of in PC-12 cells was detected using ELISA. Briefly, After the PC-12 cell were treated with capsaicin for 24 h, the supernatants from PC-12 cells were collected, and the amount of secreted 5-HT was measured using an ELISA kit (JEB-13410, JinYibai, Nanjing, China). The absorbance was measured using a microplate reader at 450 nm (MULTISCAN GO, Waltham, USA). All values were in the linear range, and readings were normalized to the total protein content.

### 16 s ribosomal RNA gene sequencing (16S rRNA)

Stool samples were frozen with liquid nitrogen, rapidly. Then stored at -80℃. After total genomic DNA was extracted, it was quantified by a microspectrophotometer (Nanodrop ONE, Thermo Fisher Scientific, Waltham, USA), and 1% agarose gel electrophoresis were applied to determine the quality of DNA. Specific amplification of the 16*S* rRNA gene was performed. Subsequently, the mixture of PCR products was purified by DNA clean beads (Vazyme, Nanjing, China). TruSeq Nano DNA LT Library Prep Kit was employed to prepare sequencing library. The library was sequenced on the Promega QuantiFluor fluorescence quantification system. Sequence denoising, operational taxonomic units (OTUs) clustering, phylogenetic and taxonomic profiling, and the analysis of alpha diversity and beta diversity were performed with QIIME2 dada2. LEfSe analysis was used to recognize bacteria of different genera. Clustering correlation heatmap with signs was performed using the OmicStudio tools at https://www.omicstudio.cn.

### Fecal microbiota transplantation

Fecal transplantation was performed in accordance with a slightly modified report [30]. Briefly, stools from the mice with 100 mg/kg capsaicin diet or CDX model mice were converged and contained at -80℃. 100 mg stools were accurately weighed and resuspended using 1 mL of sterile saline. The stool samples were thoroughly homogenized via vortexing, and centrifuged for 3 min (800 × g). The supernatants were carefully collected as the transplant materials, which were prepared and used immediately. ABX water containing ampicillin (1 g/L; CAS NO.69–52-3, Solarbio, Bejing, China), metronidazole (1 g/L; CAS NO.443–48-1, Solarbio, Bejing, China), vancomycin (0.5 g/L; CAS NO.1404–93-9, Solarbio, Bejing, China), and neomycin (0.5 g/L; CAS NO. 1405–10-3, Solarbio, Bejing, China) was supplied to mice for 5 days before transplantation. The mice were given 200 µL of the transplant materials orally once per week.

### Statistical analysis

Data were all represented as mean ± SD. GraphPad Prism 9.0 software (CA, USA) was applied to perform the statistical analyses. Unpaired Student’s test and one-way ANOVA analysis were applied for comparisons between 2 groups and 2 more groups respectively. *P* values marked in figures as: *p < 0.05, **p < 0.01, ***p < 0.001.

## Results

### Long-term intake of high-dose capsaicin orchestrates the progression of gastric cancer

**Fig. 1:**
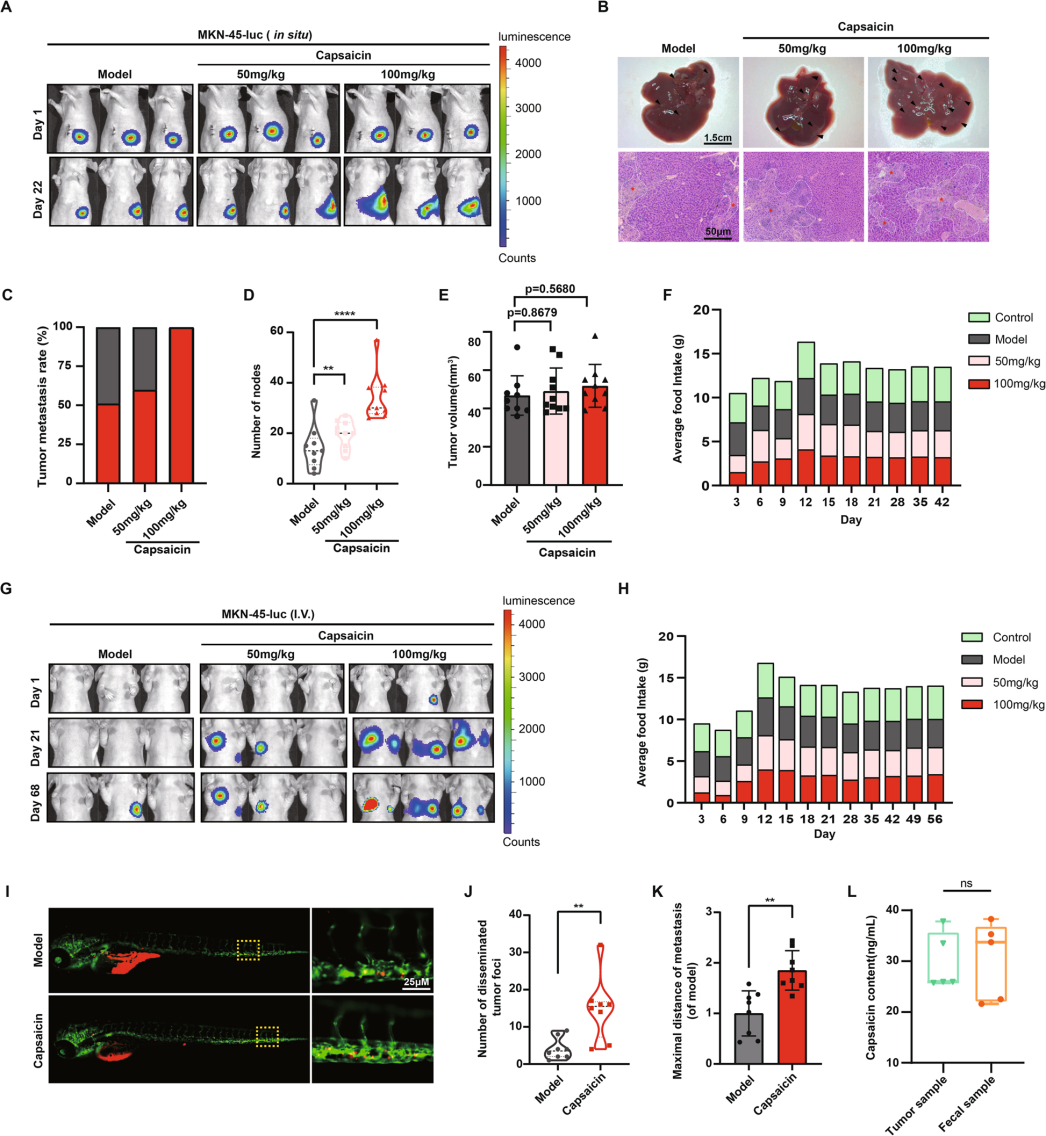
Capsaicin consumption drives metastasis of GC in the MKN-45-mediated xenograft models. **A** 1 × 10^6^ MKN-45-luc cells were injected into the epidermis of the greater curvature of the stomach in the male BALB/c nude mice. Representative bioluminescence images of mice were captured on day 1 and day 22 after the treatment of 50 mg/kg capsaicin or 100 mg/kg capsaicin. **B** Representative images of the livers (upper panel) and H&E staining (bottom panel) for liver sections (scale bars: 1.5 cm for liver images; 100 μm for H&E staining images). **C** The quantification for liver metastasis rates of the mice from model and capsaicin-treated mice. **D** The quantification for number of liver metastatic nodules, *n* = 9 for model group and *n* = 10 for the other groups. **E** The quantification for tumor volume of MKN-45 orthotropic tumors, *n* = 9 for model group and *n* = 10 for the other groups. **F** Average food intake of the tumor-bearing mice from the model group, 50 mg/kg capsaicin-treated group, and 100 mg/kg capsaicin-treated group. **G** 1 × 10.^6^ MKN-45-luc cells were injected into the caudal vein of male BALB/c nude mice. Representative bioluminescence images of mice were shown on day 1, day 21 and day 68 after the treatment of capsaicin. **H** Average food intake of the mice receiving intravenous injection of MKN-45-luc cells from the model group, 50 mg/kg capsaicin-treated group, and 100 mg/kg capsaicin-treated group. **I** Representative fluorescence (488 nm and 594 nm) images displaying the migration of Dil-labelled MKN-45 cells to the yolk sac (scale bar: 25 μm). **J** The quantification for number of disseminated Dil-labelled MKN-45 cells, *n* = 8. **K** The quantification for the maximal distances of metastasis, *n* = 8. **L** The quantification for content of capsaicin in the MKN-45 tumor samples and fecal samples, *n* = 5

In order to investigate the role of high capsaicin diet in the process of GC, 1 × 10^6^ MKN-45 GC cells were suspended in 100 μL PBS, then orthotopically injected into the greater curvature of mice. Subsequently, the mice were fed with capsaicin-containing diet (50 mg/kg or 100 mg/kg) for indicated time. The growth of primary tumors and liver metastasis were assessed accordingly. It was demonstrated that MKN-45 tumor-bearing mice treated with 100 mg/kg capsaicin displayed more metastasis than those fed with normal diet (Fig. 1A and B). Likewise, as shown in Fig. 1C and D, mice treated with 100 mg/kg capsaicin diet exhibited both higher liver metastasis rates and an increased number of liver metastatic nodules. At the same time, the volume of orthotopic tumors in capsaicin-treated mice showed no significant difference compared with normal diet-fed mice (Fig. 1E), which suggested that capsaicin does not affect the growth of gastric tumors in mice. Furthermore, we also monitored the average food intake of mice from each group, and found that food intake in the capsaicin-treated group (50 mg/kg or 100 mg/kg) was similar to that in the normal diet groups (Fig. 1F). To further explore the effect of capsaicin on GC metastasis, the MKN-45 cells were intravenously injected into the mice to allow the formation of metastasis of gastric tumors. As illustrated in Fig. 1G, IVIS bioluminescence imaging revealed that the capsaicin-treated mice (50 mg/kg or 100 mg/kg) developed much more metastases than the model group. Similarly, the average food intake of mice with capsaicin diet (50 mg/kg or 100 mg/kg) showed no difference with the mice in the model group (Fig. 1H).

Given that zebrafish has been recognized as a robust model to track fluorescence-labelled cells in vivo precisely, the MKN-45 cells were injected into the yolk of zebrafish larvae two days post fertilization, and the movement of cells was evaluated five days after the cell injection using a Leica Thunder Imaging System. Interestingly, it was observed that the migration of MKN-45 cells was significantly enhanced after the treatment of capsaicin (Fig. 1I, J and K). Moreover, UPLC-TQ-MS/MS was utilized to detect the level of capsaicin in the primary tumors and fecal samples of mice fed with 100 mg/kg capsaicin for a month, and the results showed that there was no significant difference in the concentration of capsaicin between tumor samples and fecal samples (Fig. 1L).

**Fig. 2:**
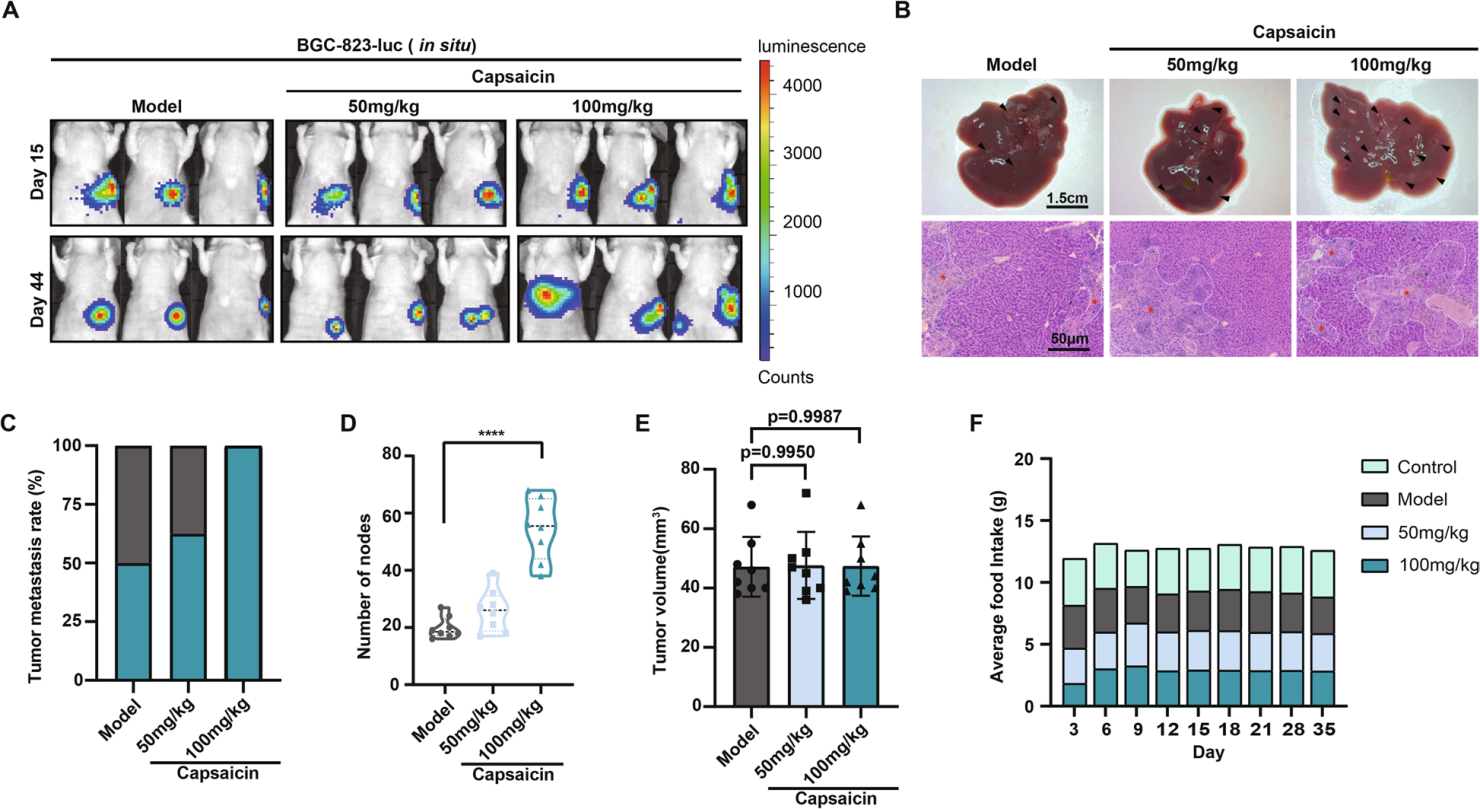
Capsaicin consumption drives metastasis of GC in BGC-823-mediated xenograft models. **A** 1 × 10^6^ BGC-823-luc cells were injected into the epidermis of the greater curvature of the stomach in the male BALB/c nude mice. Representative bioluminescence images of mice were captured on day 15 and day 44 after the treatment of capsaicin. **B** Representative images of the livers (upper panel) and H&E staining (bottom panel) for liver sections (scale bars: 1.5 cm for liver images; 50 μm for H&E staining images). The quantification for the liver metastasis rates (**C**), the number of liver metastatic nodules (**D**) and the tumor volume of BGC-823 orthotropic tumors (**E**) in the mice fed with normal diet, 50 or 100 mg/kg capsaicin, *n* = 8. **F** Average food intake of the mice receiving intravenous injection of BGC-823-luc cells from each group

The effect of high capsaicin diet on the metastasis of another GC cell type was also explored. BGC-823-luc human GC cells were injected into the greater curvature of mice. Consistently, the mice treated with capsaicin (50, 100 mg/kg) developed more hepatic metastases compared with those fed with normal diet according to the strength of luciferase intensity (Fig. 2A). This could be substantiated by the visual appearance of livers from all the groups (Fig. 2B). Additionally, H&E staining demonstrated that capsaicin contributed to increased necrotic areas of BGC-823 tumors in a dose-dependent manner (Fig. 2B). Meanwhile, both the metastasis rates (Fig. 2C) and the number of liver nodules (Fig. 2D) were remarkably augmented in the presence of 100 mg/kg capsaicin, which furtherly verified the effect of capsaicin on the metastasis of GC. Besides, the size of primary tumors (Fig. 2E) and the total food intake (Fig. 2F) remained unchanged in mice treated with capsaicin diet, compared with normal diet. In conclusion, we demonstrate that a high capsaicin diet plays a vital role in propelling the metastasis of GC though it fails to affect the growth of orthotopic gastric tumors.

### Capsaicin reinforces the motility of GC cells via instructing the activation of TRPV1

It has been well accepted that capsaicin emerges as a potent agonist of TRPV1. The capsaicin-binding pocket was generated by the S3, S4, and S4-45 linker of TRPV1, resulting in calcium influx and eliciting various pharmacological effects [31]. Furthermore, extensive literature has demonstrated the close relation between TRP protein family and the intricate process of tumor metastasis [32–34].

**Fig. 3:**
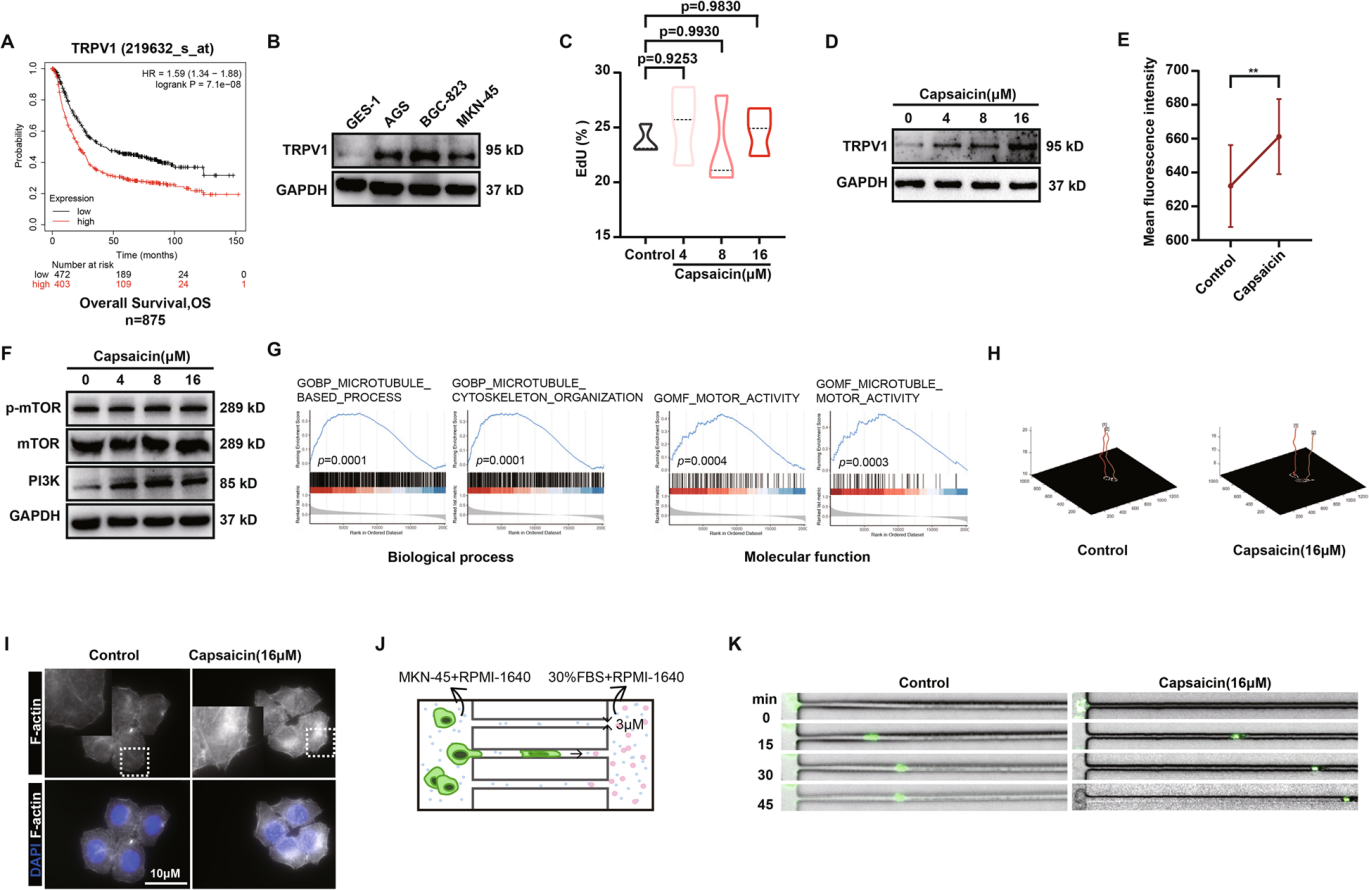
Capsaicin reinforces the motility of GC cells via instructing the activation of TRPV1. **A** Kaplan–Meier curve depicting the probability of OS for GC patients expressing high or low levels of TRPV1 (Affy ID = 219632_s_at/gene symbol = TRPV1, *n* = 875). Hazard ratio (HR) and *p*-value are shown in inset (log-rank test). **B** The protein expression of TRPV1 in the GES-1 cells, AGS cells, BGC-823 cells, and MKN-45 cells. GAPDH was used as a loading control. **C** EdU assay for the MKN-45 GC cells treated with 4 μM, 8 μM, or 16 μM capsaicin for 24 h, *n* = 3. **D** TRPV1 protein expression level in the MKN-45 GC cells treated with 4 μM, 8 μM, or 16 μM capsaicin for 24 h. **E** Mean fluorescence intensity of Ca^2+^ in the MKN-45 GC cells treated with 16 μM capsaicin for 5 s, *n* = 3. **F** The protein expression of mTOR, p-mTOR, and PI3K in the MKN-45 GC treated with 4 μM, 8 μM, or 16 μM capsaicin for 24 h. GAPDH was used as a loading control. **G** Gene Set Enrichment Analysis (GSEA) of TRPV1 expression in the GC patients showing its associations with the biological processes and molecular functions. **H** Cell Tracker was used to draw the motion trail of MKN-45 GC cells after the treatment of 16 μM capsaicin. Tracking of cell movement was shown. **I** Representative images of fluorescence staining for the actin cytoskeleton in the MKN-45 GC cells treated with 16 μM capsaicin for 24 h (scale bar: 10 μm). **J** Pattern diagram of the microfluidic chip. **K** Representative images of the MKN-45 GC cells treated with DMSO and 16μM capsaicin passing through the microfluidic chips

In this regard, we sought to investigate whether the enhanced GC metastasis induced by capsaicin was mediated via TRPV1. Kaplan–Meier plotter survival curve analysis (http://kmplot.com/analysis/index.php?p=background) was used to determine the correlation between TRPV1 expression and the overall survival (OS) of 875 GC patients. The data revealed that high expression of TRPV1 was significantly negatively correlated to the OS of GC patients (Fig. 3A). Moreover, the protein expressions of TRPV1 in several types of GC cells (AGS, BGC-823, MKN-45) were detected by immunoblotting assay. It was found that, compared with normal epithelial cells (GES-1), the protein expression of TRPV1 was strikingly boosted in the GC cells (Fig. 3B). Both CCK-8 assay (Fig. S1A and C) and EdU assay (Figs. 3C, S1B, and D) were further performed to examine the impact of capsaicin on GC cell proliferation. It was revealed that capsaicin had no significant effect on cell proliferation at concentrations under 16 μM.

Since TRPV1 protein is predominantly expressed on the cell membrane, we thus detected the expression of TRPV1 membrane protein after the treatment of capsaicin. The results elucidated that capsaicin resulted in boosted expression of TRPV1 membrane protein in a dose-dependent manner (0–16 μM) (Fig. 3D). Furthermore, capsaicin induced calcium flux was assessed in MKN-45 GC cells. It was illustrated that the fluorescent intensity of Ca^2+^ influx was dramatically elevated when exposed to capsaicin, confirming that TRPV1 can be activated upon capsaicin treatment (Fig. 3E). In line with these data, expressions of p-mTOR and PI3K, the downstream of Ca^2+^ influx, were markedly increased in the presence of capsaicin (Fig. 3F). In addition, the chip data from 375 patients with GC were analyzed using the LinkedOmics database (http://www.linkedomics.org), and the genes related to TRPV1 were excavated. Further enrichment analysis of the biological processes and molecular functions associated with these genes demonstrated that microtubule-based process, microtubule cytoskeleton organization, motor activity, microtubule motor activity, and other genes related to cell motility were positively correlated with the expression of TRPV1 (Fig. 3G). Consistently, observations tracking the movement of GC cells highlighted that capsaicin exerted prominent effects in extending the range of cell motion (Fig. 3H). Wound-healing results also showed that capsaicin treatment gave rise to the elevated migration ability of MKN-45 and BGC-823 GC cells (Fig S1E-H). Additionally, the immunofluorescence staining data elaborated that the F-actin formation of MKN-45 GC cells was profoundly potentiated when exposed to capsaicin (Fig. 3I). Moreover, the dynamic monitoring of cell movement by virtue of microfluidic chips depicted that capsaicin tended to strengthen the motility of MKN-45 GC cells in the highly confined spaces (Fig. 3J and K). Collectively, these results suggest that the effect of capsaicin in promoting GC metastasis is, at least in part, attributed to the activation of TRPV1, which consequently induces the escalated migration capability of GC cells.

### Capsaicin accelerating the progression of GC was partially mediated through the functional TRPV1

**Fig. 4:**
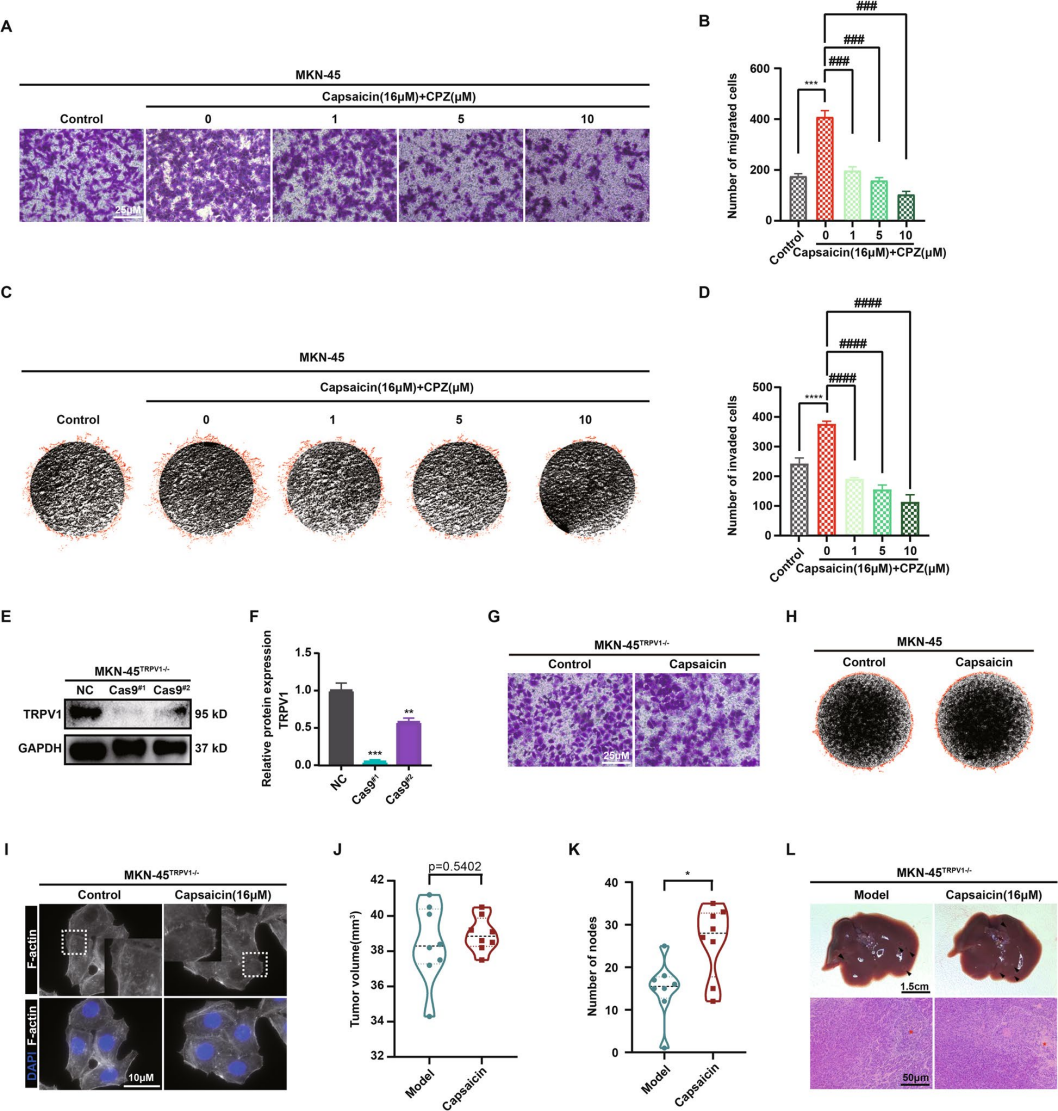
Capsaicin retarding the progression of GC was partially mediated through the functional TRPV1. Representative images (**A**) and quantitative analysis (**B**) for the migration of MKN-45 cells in the absence and presence of capsazepine (CPZ) using the transwell migration assay. Visual fields were selected randomly from each sample (scale bars: 250 μm). Representative images (**C**) and quantitative analysis (**D**) for the invasion of MKN-45 cells invasion in the absence and presence of CPZ using the 3D-invasion system (*n* = 3). Representative images (**E**) and quantitative data (**F**) for the protein level of TRPV1 in the MKN-45 cells and MKN-45^TRPV1−/−^ cells (*n* = 3). GAPDH was used as a loading control. **G** Representative images of migration of MKN-45 and MKN-45^TRPV1−/−^ cells using the transwell migration assay. Visual fields were selected randomly from each sample (scale bars: 250 μm). **H** Representative images of invasion of MKN-45 and MKN-45^TRPV1−/−^ cells using the 3D-invasion system. **I** Representative images of fluorescence staining for the actin cytoskeleton in the MKN-45^TRPV1−/−^ cells after the treatment of 16 μM capsaicin for 24 h (scale bar: 10 μm). The tumor volume (**J**) and the number of liver metastatic nodules (**K**) of MKN-45.^TRPV1−/−^ orthotropic tumors in the normal diet and 100 mg/kg capsaicin diet groups were shown. **L** Representative images for the liver (upper panel) and H&E staining sections (bottom panel) (scale bars: 1.5 cm for liver images and 50 μm for H&E-stained images)

To further explore the impact of TRPV1 on capsaicin-mediated GC metastasis, capsazepine, a recognized inhibitor of TRPV1, was employed to selectively block the function of TRPV1 prior to the treatment of capsaicin. As shown in Fig. 4A and B, the transwell migration assay results elucidated that the acceleration of vertical migration in MKN-45 GC cells induced by 16 μM capsaicin could be reversed by capsazepine in a dose-dependent manner. Homoplastically, capsazepine was also capable of dose-dependently attenuating the elevated invasion ability of MKN-45 GC cells induced by capsaicin (Fig. 4C and D). All these data in the MKN-45 GC cells were validated in the BGC-823 GC cells again, confirming the role of TRPV1 in capsaicin-mediated metastasis in GC (Fig S2C-F). In addition, TRPV1-KO-MKN-45 GC cells were generated via CRISPR/Cas9 gene-editing technology and the knockout efficiency of TRPV1 was verified by western blot (Fig. 4E and F). In agreement with above, both the transwell migration assay and 3D invasion assay uncovered that the silenced expression of TRPV1 (TRPV1-KO) remarkably prevented the impact of capsaicin in driving GC metastasis (Fig. 4G, H and Fig S2A, B). These data were in coincident with the data from immunofluorescence analysis of F-actin staining in vitro, further corroborating the role of TRPV1 in governing capsaicin-induced enhancement of GC cell migration capability (Fig. 4I).

However, we observe a range of inconsistent results in the xenograft model. For this model, TRPV1-KO-MKN-45 cells were used to establish the mouse xenograft model, followed by the treatment of 100 mg/kg capsaicin. Interestingly, the number of liver metastatic nodules was only slightly diminished in the mice receiving TRPV1-KO-MKN-45 cells compared with the mice receiving MKN-45 cells. More specifically, the average number of liver metastatic nodules in the mice receiving MKN-45 cells was around twenty (see Fig. 1D) and the mean number of liver metastatic nodules in the mice receiving TRPV1-KO-MKN-45 cells was approximately fifteen (Fig. 4K). Surprisingly, difference of tumor size between normal diet group and capsaicin-treated group was not found significant (Fig. 4J), capsaicin still tended to augment the number of liver metastatic nodules in the mice receiving TRPV1-KO-MKN-45 cells (Fig. 4K and L). Based on the results obtained from UPLC-TQ-MS/MS analysis, which showed a similar amount of capsaicin existed in both tumor and fecal samples (see in Fig. 1L), along with the regulatory role of capsaicin in gut microbiota, we thus postulated that the inconsistent results observed between in vivo and in vitro experiments might be attributed to the disruption of gut microbial homeostasis induced by capsaicin.

### Capsaicin increases peripheral 5-HT levels by modulating gut microbiota

**Fig. 5:**
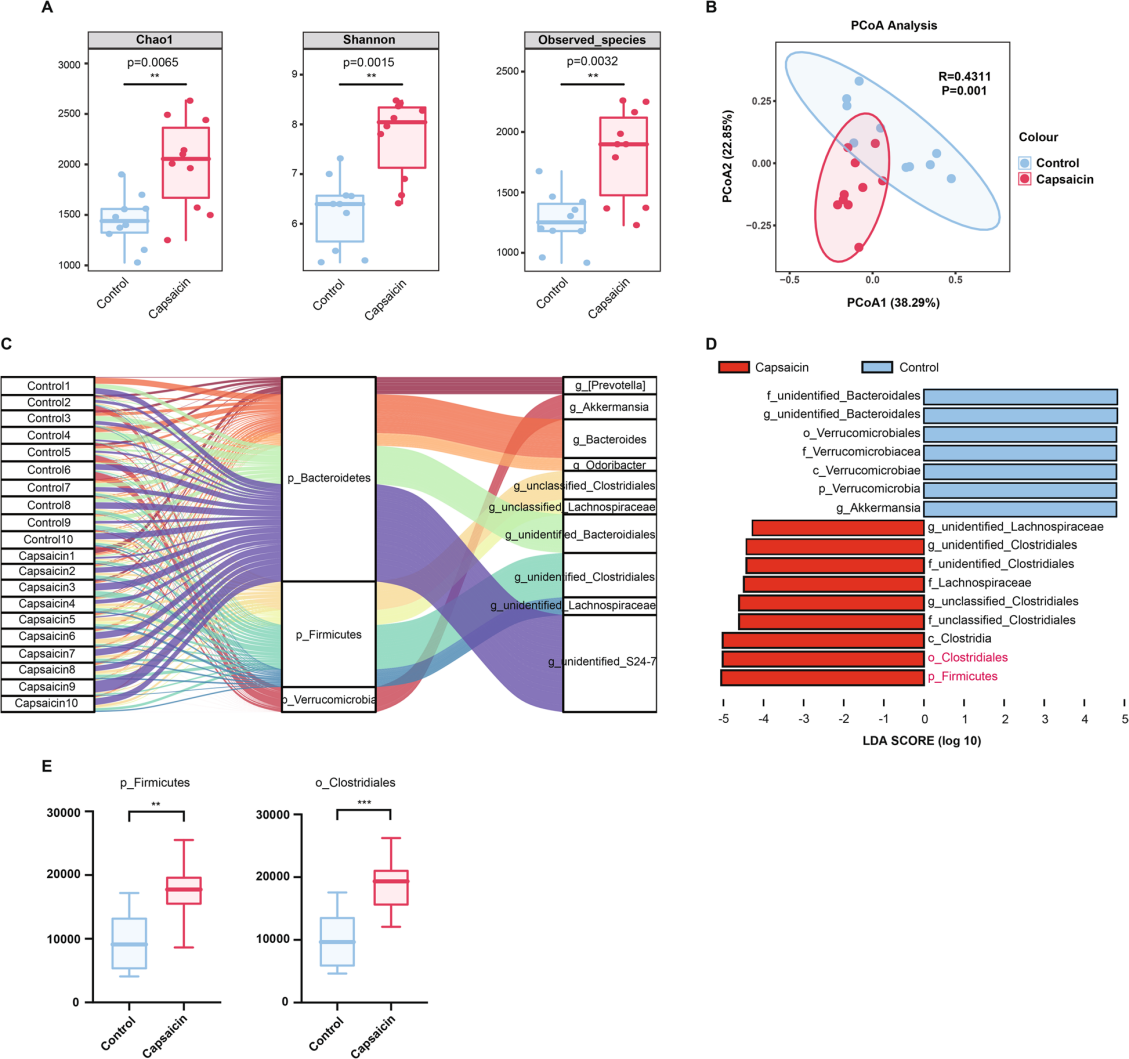
16*S* rRNA sequencing revealed changes in microbiota composition after capsaicin treatment (**A**) Alpha diversity analysis of the gut microbiota. **B** PCoA analysis of the gut microbiota. A weighted version of UniFrac-based PCoA with the PERNOVA significance test was used to generate the plot, *n* = 10. **C** Sankey plot representing the relative abundance of microbiota at the phylum level and the genus level between the normal diet and capsaicin-fed groups. **D** LDA scores derived from LEfSe analysis with Wilcoxon signed-rank test (LDA score of > 4, *p* < 0.05). **E** Relative abundance of *Firmicutes* and *Clostridiales*, *n* = 8

In order to inquire whether alterations in gut microbiota were involved in capsaicin-mediated GC metastasis, we therefore conducted 16*S* ribosomal RNA gene sequencing for bacterial identification. Three types of analysis were employed to reveal the alpha diversity in gut microbiota. Chao1 and Observed-species were used to characterize richness, and Shannon was employed to characterize diversity. Strikingly, it was demonstrated that capsaicin treatment (100 mg/kg) expressively increased the abundance and multiplicity of gut microbiota in the MKN45-xenograft mice (Fig. 5A). The PCoA revealed the microbiota composition difference between normal diet and capsaicin-treated groups. Permutational multivariate analysis of variance (PERMANOVA) exhibited conspicuous statistical differences in microbiota composition between control and 100 mg/kg capsaicin-fed groups (Fig. 5B). Subsequently, we drew the Sankey plot to describe the compositions of gut microbiota species in samples. It was illustrated that, at the phylum level, *Bacteroidetes* and *Firmicutes* occupied predominant position in the fecal samples of the two groups (Fig. 5C). To further identify the most significant differential microbiota between normal diet and 100 mg/kg capsaicin-fed groups (LDA score > 4, *p* < 0.05), we performed LEfSe analysis accordingly (Fig. 5D). Notably, it was uncovered that there was a significant increase in *Firmicutes* and *Clostridiales* among 100 mg/kg capsaicin-fed mice (Fig. 5E).

**Fig. 6:**
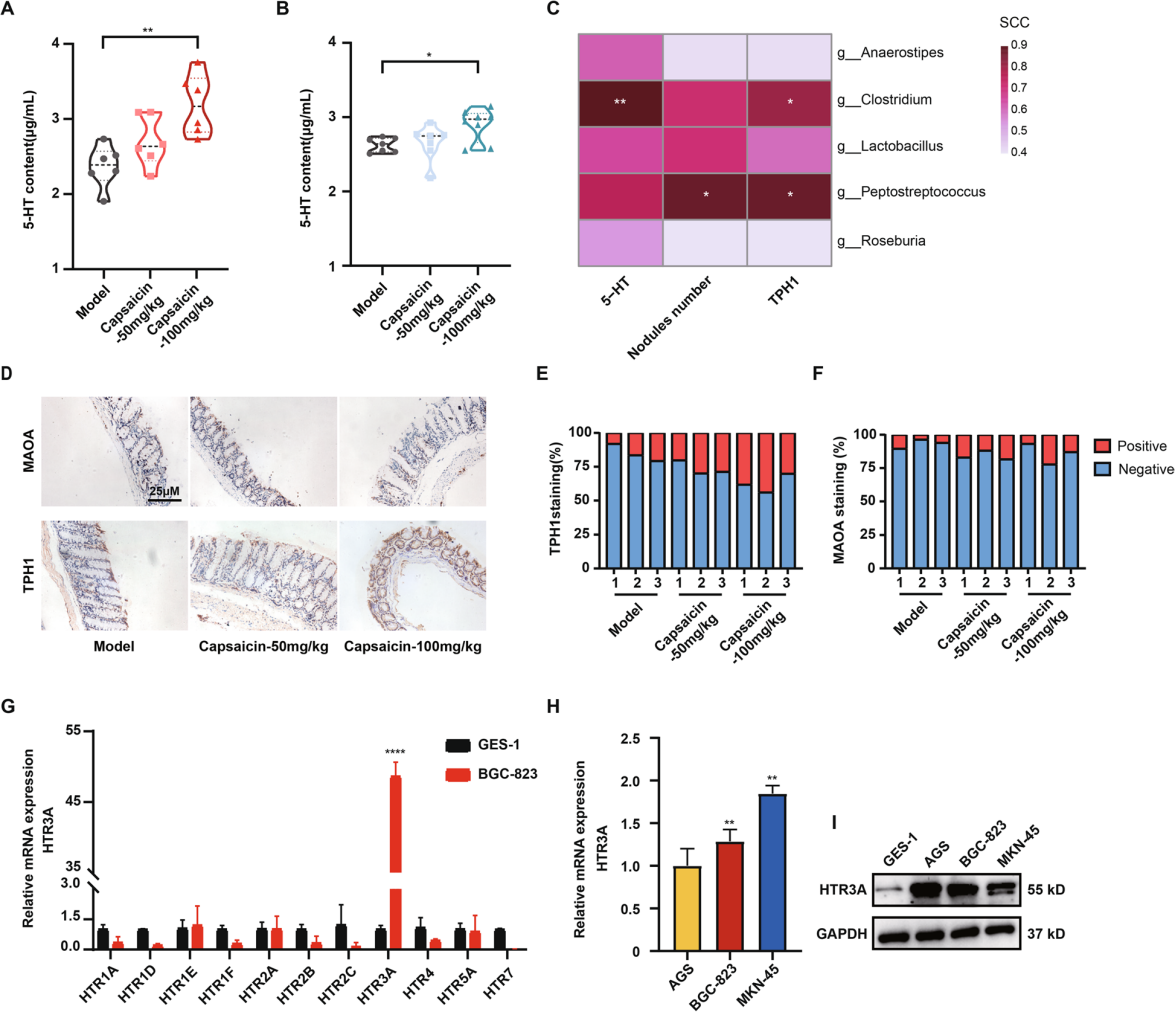
Capsaicin increases peripheral 5-HT levels. Measurement of the concentrations of 5-HT in the fecal samples from MKN-45 (**A**) and BGC-823 (**B**) xenograft mice, *n* = 6. **C** Correlation analysis of the most significantly changed microbiota upon capsaicin treatment with the expression levels of 5-HT, TPH1, as well as the number of liver metastatic nodules, *n* = 3 (Spearman’s rank correlation coefficient). **D** Representative immunohistochemistry images of MAOA and TPH1 in the colon tissues (scale bars: 25 μm). Quantitative analysis for the positive staining of TPH1 (**E**) and MAOA (**F**) in the colon tissues, *n* = 3. **G** mRNA expression levels of an array of 5-HT receptors in the BGC-823 cells relative to the GES-1 cells, *n* = 3. **H** mRNA expression levels of *HTR3A* in the BGC-823 and MKN-45 GC cells relative to the AGS cells. **I** The protein expression levels of HTR3A in the GES-1, AGS, BGC-823, and MKN-45 cell lysates. GAPDH was used as a loading control

To further dissect whether the changes in the microbiota composition induced by capsaicin were associated with the exacerbated GC progression, we performed literature reviews and found that the predominant growth of *Firmicutes* and *Clostridiales* was closely associated with the elevated peripheral 5-HT levels [35–37]. Meanwhile, the roles of peripheral 5-HT in tumorigenesis and cancer progression have been widely validated [38, 39]. To this end, we detected the content of 5-HT in the serum samples from the tumor xenograft models. Interestingly, it was observed that the treatment of 100 mg/kg capsaicin was able to increase the concentration of 5-HT in both MKN-45 and BGC-823 tumor xenograft models (Fig. 6A and B). All these analyses demonstrated a significant abundance of *Firmicutes* and *Clostridiales* after capsaicin treatment compared with normal diet. Further, the correlation analysis underscored that the significantly changed microbiota by capsaicin treatment was positively correlated to the expression levels of 5-HT and TPH1, as well as the number of metastatic nodules in the liver (Fig. 6C). Considering that the majority of chromaffin cells, responsible for peripheral 5-HT production, are located in the colon, as well as that TPH1 and MAOA are served as two critical rate-limiting enzymes involved in 5-HT synthesis and degradation, respectively, we thus detected the expression levels of TPH1 and MAOA in colon tissues (Fig. 6D-F). Intriguingly, our data elucidated that the treatment of 100 mg/kg capsaicin boosted the protein expression level of TPH1 whereas it had no significant impact on the protein expression of MAOA, implying that capsaicin presumably influenced the concentration of peripheral 5-HT through enhancing the protein expression of TPH1. Additionally, we also measured the expression levels of 5-HT receptors in the GC cells, as 5-HT functions via binding to its receptor. We initially examined the mRNA expression of multiple 5-HT receptors that have been confirmed to involve in tumor progression, and it was found that HTR3A was significantly overexpressed in the GC cells compared with GES-1 gastric epithelial cells (Fig. 6G and H). The protein expression levels of HTR3A in various types of GC cells were further assessed. The results proved that HTR3A protein expressions in the GC cells (AGS, BGC-823 and MKN-45) were significantly higher than that in the GES-1 cells (Fig. 6I). The above data confirmed that the impact of capsaicin on gut microbiota and altered microbiota composition may further propel GC metastasis via regulating the process of 5-HT synthesis and the subsequent interactions between 5-HT and its receptor HTR3A.

### HTR3A is involved in 5-HT-mediated metastasis of GC in response to capsaicin treatment

**Fig. 7:**
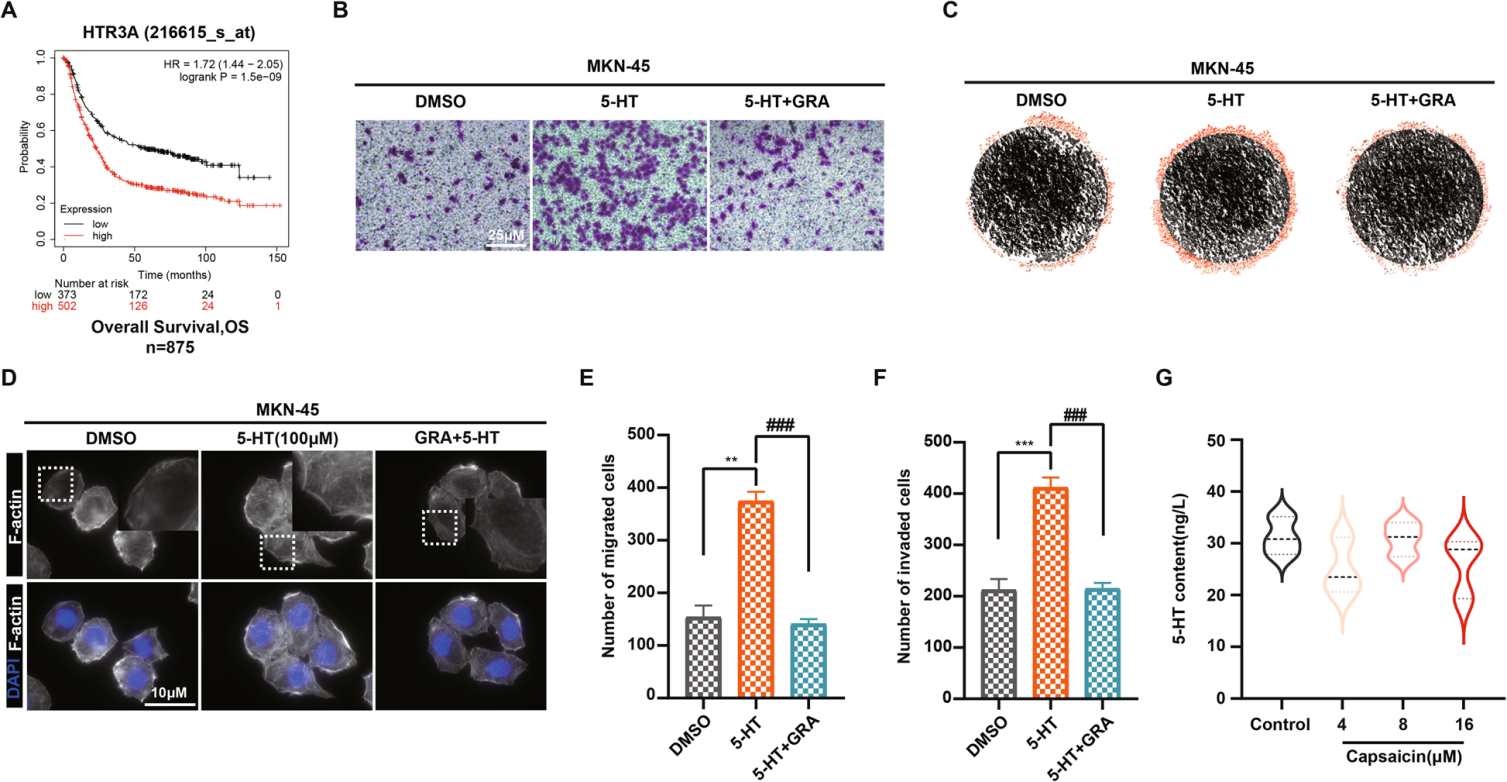
HTR3A is involved in 5-HT-mediated metastasis of GC in response to capsaicin treatment. **A** Kaplan–Meier curve depicting the probability of OS for GC patients expressing high or low levels of *HTR3A*. (Affy ID = 216615_s_at/gene symbol = *HTR3A*, *n* = 875). Hazard ratio (HR) and *p*-value were shown in inset (log-rank test). **B** Representative images of the migration of MKN-45 GC cells with or without pre-incubation with granisetron for 30 min using the transwell system. Visual fields were selected randomly from each sample (scale bars: 25 μm). **C** Representative images of the invasion of MKN-45 GC cells with or without pre-incubation with granisetron for 30 min using the 3D-invasion system. **D** Representative images of fluorescence staining for the actin cytoskeleton in the MKN-45 GC cells with or without pre-incubation with granisetron for 30 min (scale bar: 10 μm). The quantitative data for the migration (**E**) and invasion (**F**) of MKN-45 GC cells with or without pre-incubation with granisetron for 30 min, *n* = 3. **G** The quantification for the concentration of 5-HT from the PC-12 cells upon capsaicin treatment

To uncover whether HTR3A participate in the GC progression, a Kaplan–Meier plotter survival curve analysis between the OS of GC patients and HTR3A expression was carried out. It was shown that the higher expression of HTR3A, the shorter OS of those GC patients. In other words, HTR3A expression was negatively corresponded to the OS of GC patients (Fig. 7A). To further assess the potential impact of 5-HT binding to HTR3A in driving GC metastasis, we proceeded to stimulate GC cells with 5-HT in order to elucidate its effects on the migration and invasion capabilities of GC cells. Our data unveiled that 5-HT stimulation reinforced the migration and invasion of GC cells, whereas the increased migration and invasion induced by 5-HT was remarkably reversed following the pre-incubation with the HTR3A-specific antagonist granisetron (Fig. 7B, C and E, F). The immunofluorescence staining for F-actin in the GC cells demonstrated that granisetron reversed 5-HT-mediated migration and invasion in MKN-45 cells probably through suppressing F-actin aggregation (Fig. 7D). In addition, the concentration of 5-HT in the supernatants of PC-12 cells were examined after 24 h treatment of capsaicin, and it was observed that capsaicin failed to display direct effect on stimulating 5-HT secretion in the chromaffin cells (Fig. 7G), implying that capsaicin exerts indirect effect on 5-HT secretion via modulating gut microbiota. Overall, these results suggest that overexpressed expression of HTR3A in GC cells is involved in capsaicin sustaining GC metastasis through modulation of gut microbiota.

### Fecal microbiota derived from high dose of capsaicin-fed mice accelerates the GC metastasis

**Fig. 8:**
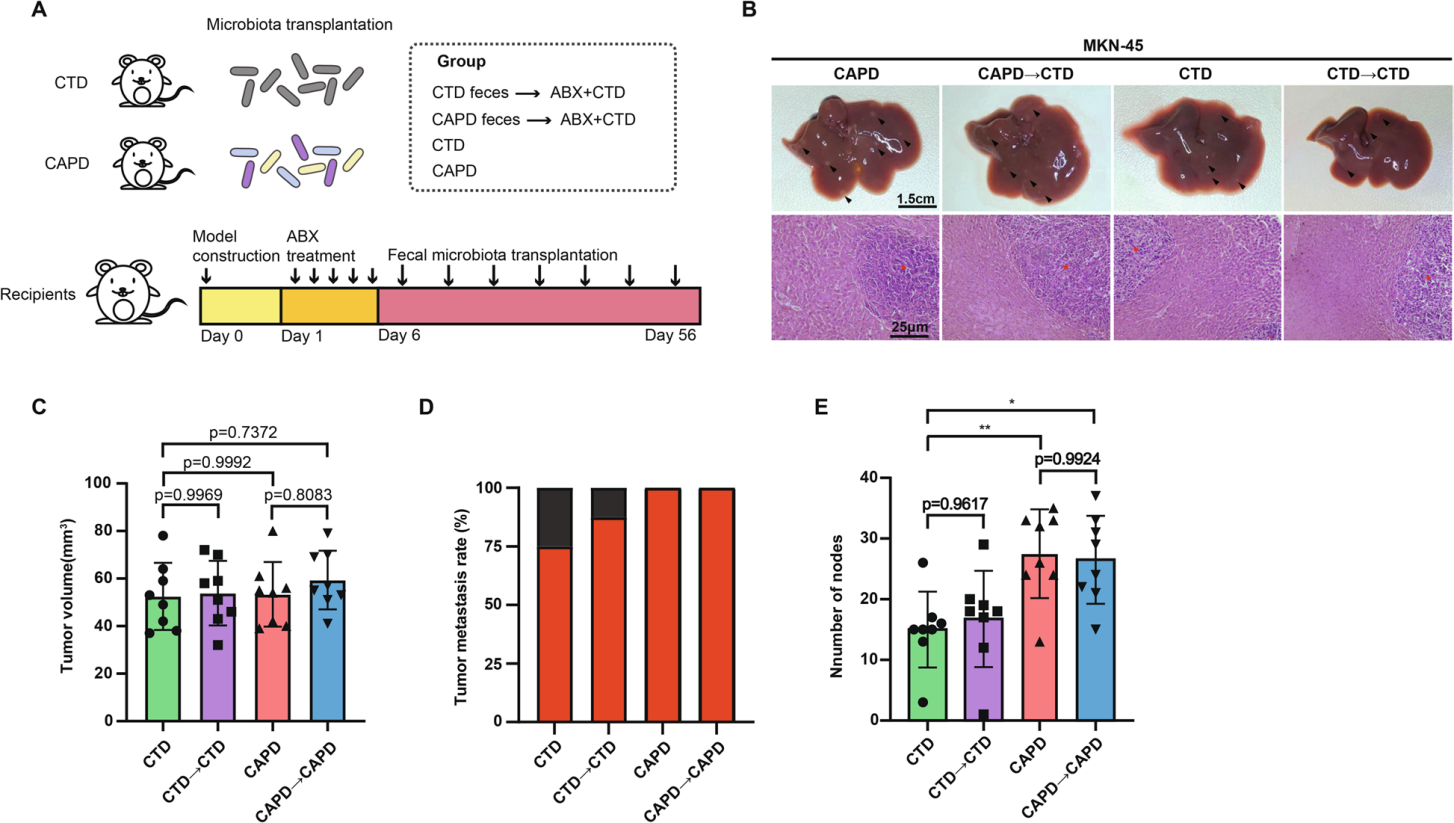
Fecal microbiota derived from CAPD mice accelerates the metastasis of GC. **A** Schematic diagram of the FMT procedure. **B** Representative images of the liver (upper panel) and H&E staining images (bottom panel) of the liver sections (scale bars: 1.5 cm for liver images and 25 μm for H&E staining images). The tumor volume (**C**) and liver metastasis rates (**D**) of MKN-45-luc orthotropic tumors were determined for CTD, CTD to CTD, CAPD, or CAPD to CAPD groups, *n* = 8. **E** The number of liver metastatic nodules was quantified, *n* = 8

To verify whether the disruption of microbiota composition caused by capsaicin was a driving force curbing GC metastasis, we therefore inquired into the impression of fecal samples from the capsaicin-fed mice on metastasis of GC by virtue of FMT experiment. After the establishment of tumor xenograft model, the mice were randomly divided into four groups, including control diet group (CTD), capsaicin diet donor group (CAPD), capsaicin diet recipient group (CAPD to CTD), and control diet recipient group (CTD to CTD). The mice in the recipient group were treated with feces from the mice in the donor group to examine GC progression (Fig. 8A). Intriguingly, liver histopathology results elucidated that the mice in CAPD group harbored similar liver necrotic areas compared with the CAPD to CTD group, which were significantly boosted than the mice in the CTD as well as in the CTD to CTD group (Fig. 8B). The size of tumors remained unchanged (Fig. 8C), but the tumor metastasis rates and the number of metastatic nodules in the liver were markedly augmented in the CAPD compared with the CTD group. Similar results were obtained from the CAPD to CAPD group compared with the CTD to CTD group (Fig. 8D and E). In summary, these results indicate that fecal microbiota from CAPD mice accelerates the metastasis of GC.

## Discussion

GC is deemed to be a significant contributor to the global burden of cancer [40]. It has been accepted that *Helicobacter pylori* screening and the successful development of targeted drugs for GC (e.g., Ramucirumab and Trastuzumab) are still the predominant strategies for diminishing the incidence and mortality of GC. But, a growing body of evidence in the last decades also demonstrated that diet could serve as one of the decisive factors for cancer incidence [41]. In this perspective, breaking poor dietary habits and developing healthy dietary habits are essential for attenuating cancer risk or incidence.

Food is recognized as one of the driving forces for human evolution, meanwhile, alterations in eating habits and diet structure tend to directly influence human development and health [42–44]. Chili pepper is one of the most commonly used spices globally and capsaicin is the major ingredient of it. The analgesic and metabolic modulation of capsaicin have been widely determined [45, 46], yet its role in tumor progression remains a subject of controversy. A few experimental studies showed that capsaicin could suppress the growth of GC cells or inhibit metastatic activity [47, 48]. However, many epidemiological studies have identified that a high capsaicin diet is positively correlated with an increased risk of GC [7, 12–14]. Additionally, capsaicin is commonly employed to induce GC in combination with *Helicobacter pylori* [8, 49, 50]. Hence, gaining insight into the role of the high capsaicin diet in GC progression holds immense importance in guiding the dietary habits of individuals with existing GC.

Akagi et al. reported no carcinogenic activity in B6C3F1 mice fed a basal diet supplemented with 0.025, 0.083 and 0.25% capsaicinoids for 79 weeks, in which statistical effect on GC was missed [51]. In contrast, Toth et al. reported that 0.03125% capsaicin diet significantly induced cecum tumorigenesis in Swiss mice [52]. The carcinogenic effects on GC of lifelong capsaicin administration are still up for debate. Differ from these investigations into the effect of capsaicin on tumorigenesis, our experiment aims to elucidate the role of a high capsaicin diet in tumor metastasis. In this study, effects of the high capsaicin diet on GC metastasis were confirmed from in vivo. It was demonstrated that MKN-45 GC-bearing mice with a 100 mg/kg capsaicin diet for about 2 months showed significantly elevated metastasis compared with those with a normal diet, which was supported by the dramatically increased tumor metastasis rate and the number of liver metastatic nodules in capsaicin diet groups. These results were in line with the BGC-823 GC-bearing model, indicating that the high capsaicin diet is closely associated with GC metastasis. Of note, the growth of gastric tumor in the mice fed with 100 mg/kg capsaicin remained unchanged compared with that in the mice fed with normal diet. All these data imply that the treatment of capsaicin gives rise to the increased migration and invasion abilities of GC cells and/or yields the special microenvironment that facilitates the metastasis of GC cells.

To explore the mechanism of capsaicin in promoting GC metastasis, we initially explored the direct actions of capsaicin on GC migration and invasion, and it was demonstrated that the migration and invasion of GC cells were significantly diminished based on multiple in vitro models. Indeed, previous studies uncovered that capsaicin could induce cell cycle arrest in various types of tumor cells [53, 54]. Although we also observed a similar phenomenon in the GC cells, the death of GC cells only occurred when exposed to high doses of capsaicin (> 16 μM, Fig. S1 A-D). Wu et al.reported that the treatment of capsaicin with concentrations above 50 μM capsaicin inhibited the viability of MDA-MB-231 breast cancer cells [55], suggesting that the treatment of high dosages of capsaicin leads to cancer cell death. Inversely, we demonstrated that low dosages of capsaicin had no significant effect on the proliferation of GC cells, whereas the migration and invasion of GC cells were markedly elevated. We also determined the function of capsaicin in the growth of gastric tumor, and the results showed that the direct effect of capsaicin on tumor growth was comparable to that in vitro.

Given that capsaicin is a well-accepted agonist of TRPV1, we inevitably speculated whether direct activation of TRPV1 by capsaicin promoted GC metastasis. According to the Kaplan–Meier plotter survival curve analysis, it was observed that TRPV1 expression was negatively correlated to the OS of GC patients. More interestingly, the elevated migration and invasion capabilities of capsaicin in the GC cells was profoundly reversed in the presence of CPZ that is deemed to be a classic antagonist of TRPV1, shadowing that TRPV1 acts a pivotal role in mediating the metastasis of GC in response to capsaicin. However, in the absence of TRPV1, the treatment of 100 mg/kg capsaicin in the GC mouse xenograft models still to some extent resulted in increased GC metastasis, implying that there might be other possible underlying mechanisms contributing to GC metastasis in vivo synergistically. All these data suggest that capsaicin sustains metastasis at least partially due to the activation of TRPV1.

Meanwhile, we found that the amount of capsaicin in fecal samples was comparable to that in tumor tissues. Another important aspect of our study was that capsaicin had significant impacts on gut microbiota composition, which further led to a conspicuous increase in the content of peripheral 5-HT. It has been well known that a vast and diverse group of microbes colonizing in mammals exert striking effects on health and disease. It was demonstrated that the abundance of *Clostridiales* and *Firmicutes*, which are contributing to the production of peripheral 5-HT, were significantly upregulated by capsaicin. It has been widely reported that 5-HT or its corresponding metabolites can accelerate multiple stages of tumor progression [38]. It was revealed that capsaicin contributed to increase content of peripheral 5-HT, and the interactions between 5-HT and HTR3A in the GC cells further boosted the GC metastasis. Further, FMT experiment also validated the character of gut microbiota in communicating capsaicin-promoted metastasis of GC. Notably, it was uncovered that the silence of TRPV1 prominently attenuated the elevated metastasis of GC cells in response to capsaicin in vitro, whereas the loss of TRPV1 failed to achieve similar phenomena in vivo. As such, we believe that disruption of gut microbiota results in more overt imbalance than individual proteins in the homeostasis of human body in tumor progression.

## Conclusion

In summary, our data highlight that the high capsaicin diet is a potential risk for accelerating the progression of GC metastasis. The abnormal expression and activation of TRPV1 in GC cells and the disruption of the intestinal microbial composition may serve as the key determinants that capsaicin drives GC metastasis. Indeed, capsaicin-activated TRPV1 in the GC cells gives rise to the polymerization of cytoskeletal proteins, propels the motility of GC cells, and fuels the process of metastasis of GC. Meanwhile, capsaicin alters the composition of gut microbes and increases the content of peripheral 5-HT that further binding to HTR3A on the GC cells. All of these contribute to the augmented motility of GC cells and accelerated GC metastasis. Taken together, our study suggest that the consumption of capsaicin should be controlled within an appropriate range, and targeting TRPV1 or specific microbiota offers a potential and promising therapeutic strategy for GC.

### Supplementary Information

**Additional file 1: Figure S1.** Effect of capsaicin on proliferation and migration of gastric cancer cells. (A) MKN-45 cells proliferation and viability were detected by the CCK-8 method after various doses of capsaicin treatment (*n* = 3). (B) Representative images of EdU assay of MKN-45 cells treated with 4μM, 8μM, or 16μM capsaicin for 24h (scale bar: 125μm). (C) BGC-823 cells proliferation and viability were detected by the CCK-8 method after various doses of capsaicin treatment (*n* = 3). (D) Representative images of EdU assay of BGC-823 cells treated with 4μM, 8Mm, or 16μM capsaicin for 24h (scale bar: 125μm). (E) and (G) Measurement of MKN-45 and BGC-823 cell migration ability using a wound-healing assay with or without incubating with capsaicin for 24h. Views (10×) were selected randomly from each sample. (F) and (H) Quantitative evaluation of (E) and (G) (*n* = 3). **Figure S2.** The role of TRPV1 in capsaicin-induced gastric cancer metastasis. (A) and (B) Quantitative evaluation of Fig. 3 (G) and (H). (*n* = 3). (C) Measurement of BGC-823 cell migration ability using the trans-well system with or without pre-incubating with capsazepine for 30min. Views were selected randomly from each sample (scale bars: 25μm). (D) Quantitative evaluation of (C) (*n* = 3). (E) Measurement of BGC-823 cell invasion ability using 3D-invasion system with or without pre-incubating with capsazepine for 30min. (F) Quantitative evaluation of (E) (*n* = 3). **Table S1.** Oligo sequences of TRPV1 plasmid.** Table S2.** Primer sequences used in this paper. **Table S3.** Multiple reactions monitoring (MRM) parameters of capsaicin by UPLC-TQ-MS. **Table S4.** Linear regression data of capsaicin by UPLC-TQ-MS.

## Data Availability

The raw data of 16*S* rRNA gene sequencing used in the manuscript can be obtained from NCBI (Accession number: PRJNA830971).

## Abbreviations

GC: Gastric cancer
CAP: Capsaicin
TRPV1: Transient receptor potential cation channel subfamily v member 1
CPZ: Capsazepine
HPF: Hours post-fertilization
FBS: Fetal bovine serum
CDX: Cell line-derived xenograft
OD: Optical density
OS: Overall survival
FMT: Fecal microbiota transplantation
ABx: Antibiotics
PERMANOVA: Permutational multivariate analysis of variance
LDA: Linear discriminant analysis
PCoA: Principal coordinates analysis
