# Supplementary material for: Capsaicin orchestrates metastasis in gastric cancer via modulating expression of TRPV1 channels and driving gut microbiota disorder
Source: Cell Commun Signal. 2023 Dec 21;21:364. doi: 10.1186/s12964-023-01265-3 (PMC10734064; doi:10.1186/s12964-023-01265-3)
Supplement: Supplementary file 2 — Additional file 1: Figure S1. Effect of capsaicin on proliferation and migration of gastric cancer cells. (A) MKN-45 cells proliferation and viability were detected by the CCK-8 method after various doses of capsaicin treatment (n = 3). (B) Representative images of EdU assay of MKN-45 cells treated with 4μM, 8μM, or 16μM capsaicin for 24h (scale bar: 125μm). (C) BGC-823 cells proliferation and viability were detected by the CCK-8 method after various doses of capsaicin treatment (n = 3). (D) Representative images of EdU assay of BGC-823 cells treated with 4μM, 8Mm, or 16μM capsaicin for 24h (scale bar: 125μm). (E) and (G) Measurement of MKN-45 and BGC-823 cell migration ability using a wound-healing assay with or without incubating with capsaicin for 24h. Views (10×) were selected randomly from each sample. (F) and (H) Quantitative evaluation of (E) and (G) (n = 3). Figure S2. The role of TRPV1 in capsaicin-induced gastric cancer metastasis. (A) and (B) Quantitative evaluation of Fig. 3 (G) and (H). (n = 3). (C) Measurement of BGC-823 cell migration ability using the trans-well system with or without pre-incubating with capsazepine for 30min. Views were selected randomly from each sample (scale bars: 25μm). (D) Quantitative evaluation of (C) (n = 3). (E) Measurement of BGC-823 cell invasion ability using 3D-invasion system with or without pre-incubating with capsazepine for 30min. (F) Quantitative evaluation of (E) (n = 3). Table S1. Oligo sequences of TRPV1 plasmid. Table S2. Primer sequences used in this paper. Table S3. Multiple reactions monitoring (MRM) parameters of capsaicin by UPLC-TQ-MS. Table S4. Linear regression data of capsaicin by UPLC-TQ-MS. [file 12964_2023_1265_MOESM1_ESM.docx]

# Supplementary materials


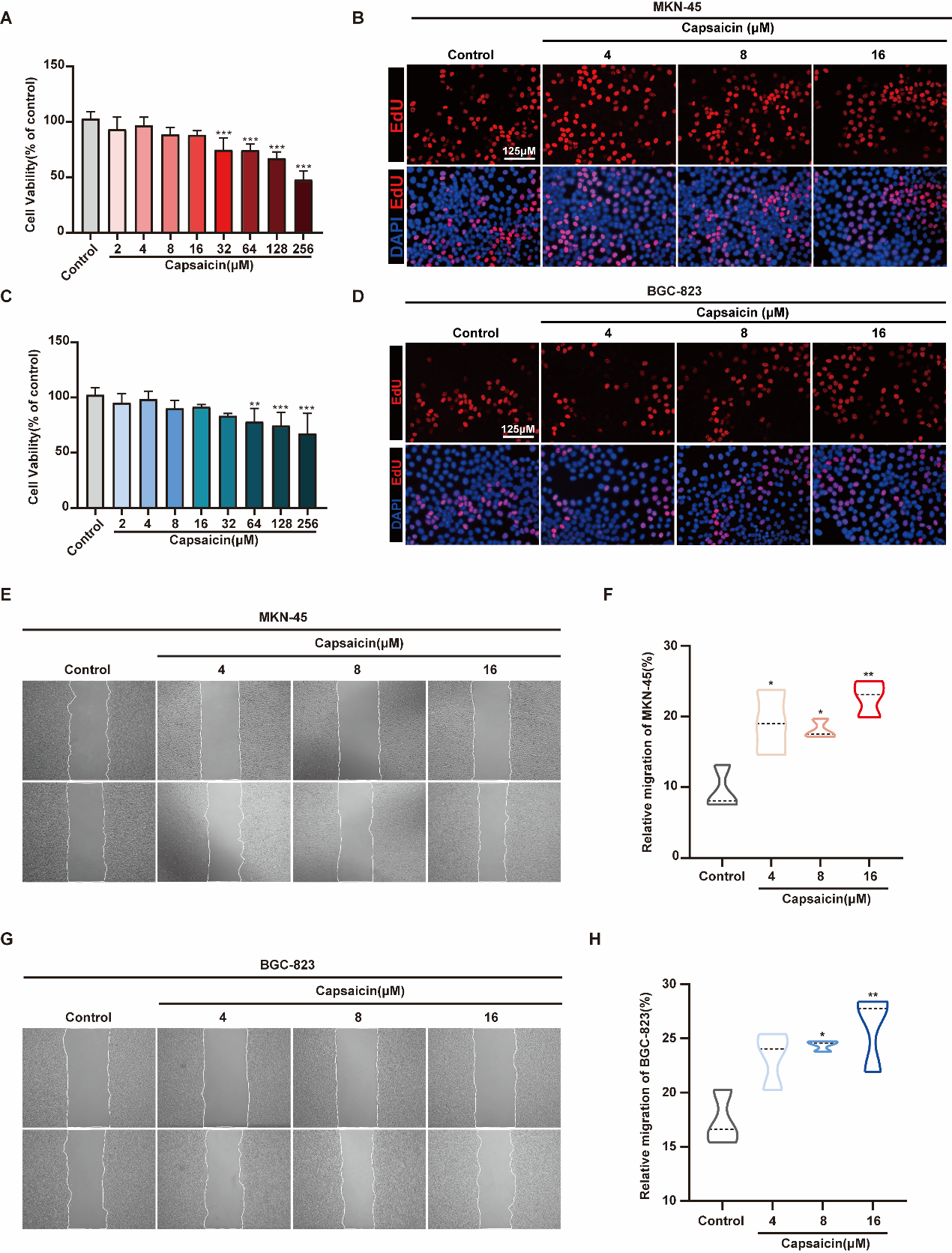


**Figure S1.** Effect of capsaicin on proliferation and migration of gastric cancer cells. (A) MKN-45 cells proliferation and viability were detected by the CCK-8 method after various doses of capsaicin treatment (n = 3). (B) Representative images of EdU assay of MKN-45 cells treated with 4μM, 8μM, or 16μM capsaicin for 24h (scale bar: 125μm). (C) BGC-823 cells proliferation and viability were detected by the CCK-8 method after various doses of capsaicin treatment (n = 3). (D) Representative images of EdU assay of BGC-823 cells treated with 4μM, 8Μm, or 16μM capsaicin for 24h (scale bar: 125μm). (E) and (G) Measurement of MKN-45 and BGC-823 cell migration ability using a wound-healing assay with or without incubating with capsaicin for 24h. Views (10×) were selected randomly from each sample. (F) and (H) Quantitative evaluation of (E) and (G) (n = 3).


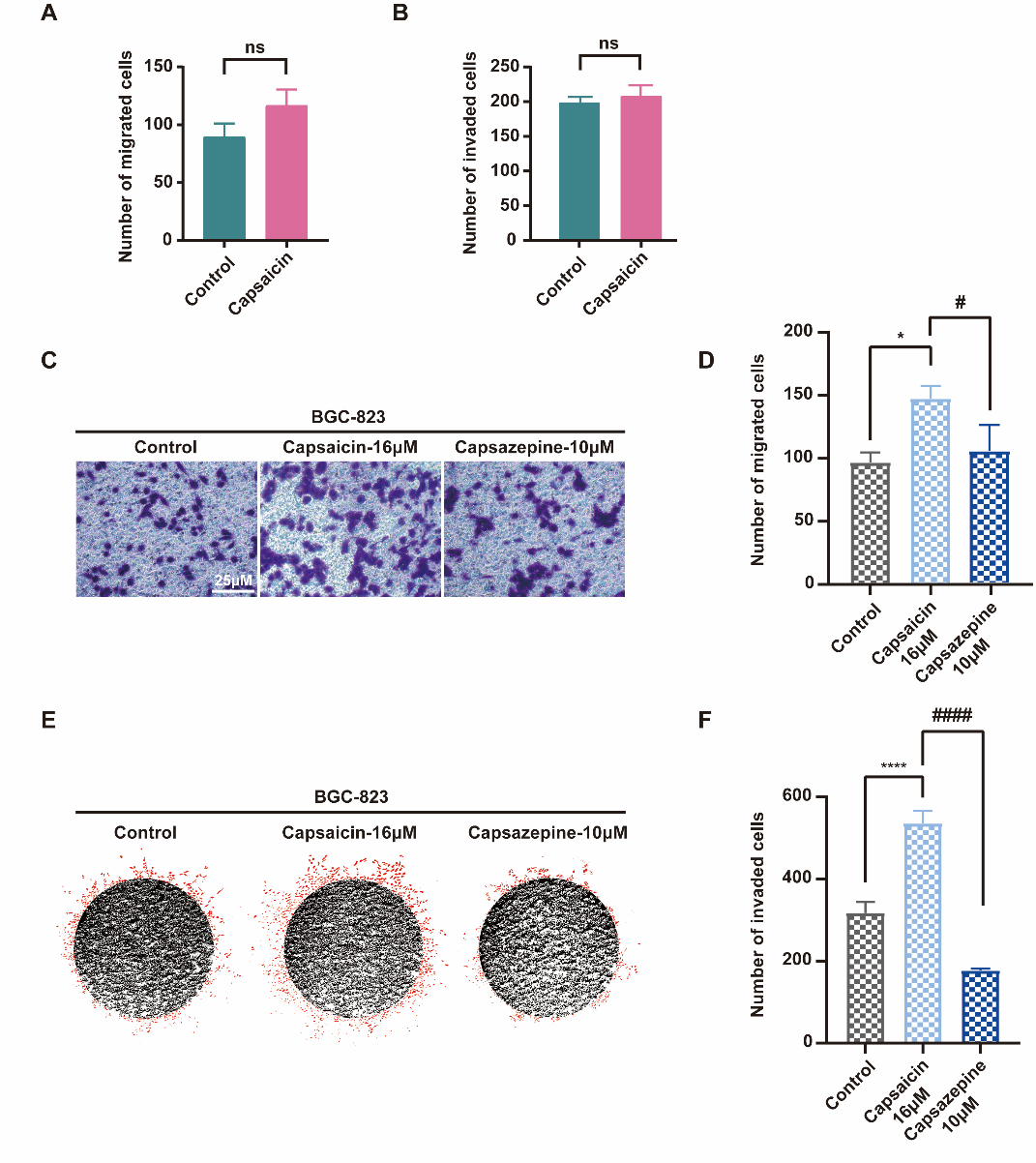


**Figure S2.** The role of TRPV1 in capsaicin-induced gastric cancer metastasis.

(A) and (B) Quantitative evaluation of Figure 3 (G) and (H). (n = 3). (C) Measurement of BGC-823 cell migration ability using the trans-well system with or without pre-incubating with capsazepine for 30min. Views were selected randomly from each sample (scale bars: 25μm). (D) Quantitative evaluation of (C) (n = 3). (E) Measurement of BGC-823 cell invasion ability using 3D-invasion system with or without pre-incubating with capsazepine for 30min. (F) Quantitative evaluation of (E) (n = 3).

**Table S1.** Oligo sequences of TRPV1 plasmid.

|  | **Oligo sequence** |
| --- | --- |
| *TRPV1*-KO1F | caccGACGGCCGACATCAGCGCCA |
| *TRPV1*-KO1R | aaacTGGCGCTGATGTCGGCCGTC |
| *TRPV1*-KO2F | caccGTTGTCGGCCACCTCCACCA |
| *TRPV1*-KO2R | aaacTGGTGGAGGTGGCCGACAAC |
| *TRPV1*-KO3F | aaacTGGTGGAGGTGGCCGACAAC |
| *TRPV1*-KO3R | aaacGCTTCAGCGTCGGGTGCAGTc |

**Table S2.** Primer sequences used in this paper.

|  | **Primer sequence** |
| --- | --- |
| *HTR3A*-Forward primer | 5’ - GAAGCCAACCACCGTATCCAT - 3’ |
| *HTR3A*-Reverse primer | 5’ - CCACATCCACGAACTCATTGAT - 3’ |
| *HTR1A*-Forward primer | 5’ - TGTATCAGGTGCTCAACAAGTG - 3’ |
| *HTR1A*-Reverse primer | 5’ - AGGAAGCCAATAAGCCAAGTG - 3’ |
| *HTR1D*-Forward primer | 5’ - GGCCAAATCTTGTGTGACATCT - 3’ |
| *HTR1D*-Reverse primer | 5’ - CGTCCTGCGTTTACTGTATTCC - 3’ |
| *HTR1E*-Forward primer | 5’ - CAATGCTATTGAATACGCCAGGA - 3’ |
| *HTR1E*-Reverse primer | 5’ - GAGATGGTCCAGACGGTAAGG - 3’ |
| *HTR1F*-Forward primer | 5’ - ACTTGACCTCAGAGGAACTGT - 3’ |
| *HTR1F*-Reverse primer | 5’ - ATTGCAGCGATCACAAGGGAG - 3’ |
| *HTR2A*-Forward primer | 5’ - CTTTGTGCAGTCTGGATTTACCT - 3’ |
| *HTR2A*-Reverse primer | 5’ - ACTGATATGGTCCAAACAGCAAT - 3’ |
| *HTR2B*-Forward primer | 5’ - ACGACTCTACAACTACGGCAG - 3’ |
| *HTR2B*-Reverse primer | 5’ - GCCAAACACTCAAAAGCCAAAG - 3’ |
| *HTR2C*-Forward primer | 5’ - TCAATACCTCCGATGGTGGAC - 3’ |
| *HTR2C*-Reverse primer | 5’ - GGTGGCATTGTGCAGTTTCTT - 3’ |
| *HTR4*-Forward primer | 5’ - CTCACGTTTCTCTCGACGGTT - 3’ |
| *HTR4*-Reverse primer | 5’ - AGCAGATCCGCAAAAGCAAGA - 3’ |
| *HTR5A*-Forward primer | 5’ - CACGCGCCACATGGAATAC - 3’ |
| *HTR5A*-Reverse primer | 5’ - CCCTCAGAGTACGTCTCTCC - 3’ |
| *HTR6*-Forward primer | 5’ - GCAACACGTCCAACTTCTTCC - 3’ |
| *HTR6*-Reverse primer | 5’ - TGCAGCACATCACGTCGAA - 3’ |
| *HTR7*-Forward primer | 5’ - CGAAGATGATTCTCTCCGTCTG - 3’ |
| *HTR7*-Reverse primer | 5’ - GCGGTAGAGTAAATCGTATAGCC - 3’ |
| *GAPDH*-Forward primer | 5’ - GGTTGTCTCCTGCGACTTCA - 3’ |
| *GAPDH*-Reverse primer | 5’ - TGGTCCAGGGTTTCTTACTCC - 3’ |

**Table S3.** Multiple reactions monitoring (MRM) parameters of capsaicin by UPLC-TQ-MS.

| **Compounds** | **Ion mode** | **Precursor/Production** | **CE (eV)** | **DP (V)** | **CXP (V)** |
| --- | --- | --- | --- | --- | --- |
| Capsaicin | [M+H]^+^ | 306.2/244.2 | 27.0 | 1.0 | 22.0 |

**Table S4.** Linear regression data of capsaicin by UPLC-TQ-MS.

| **Analyte** | **Regression equation** | **Linear range (ng/mL)** | **R** |
| --- | --- | --- | --- |
| Capsaicin | $y=43723x+15833$ | 2.6-42.2 | 0.9989 |
